# Exploring the Antifibrotic Mechanisms of Ghrelin: Modulating TGF-β Signalling in Organ Fibrosis

**DOI:** 10.1017/erm.2024.38

**Published:** 2024-11-21

**Authors:** Mei Li, Chang Zheng, Huiyi Wang, Shan Wang

**Affiliations:** Department of Oral Pathology, School of Stomatology, Hainan Medical University, Haikou, P. R. China

**Keywords:** antifibrotic mechanisms, fibrosis, ghrelin, organ, TGF-β

## Abstract

**Background:**

Fibrosis is a pathological condition that affects various organs by increasing fibrous connective tissue while reducing parenchymal cells. This imbalance can lead to compromised organ function and potential failure, posing significant health risks. The condition’s complexity necessitates the exploration of effective treatments to mitigate its progression and adverse outcomes.

**Aims:**

This study aims to investigate the role of ghrelin, a peptide hormone known for its anti-inflammatory and anti-fibrotic properties, in modulating fibrosis across different organs. By binding to the growth hormone secretagogue receptor type 1a (GHSR-1a), ghrelin has shown potential in attenuating the fibrotic process, particularly through its interaction with the TGF-β signalling pathway.

**Methods:**

An extensive review of clinical and animal model studies focusing on liver, kidney, lung, and myocardial fibrosis was conducted. The primary focus was on examining how ghrelin influences the TGF-β signalling pathway, with an emphasis on the regulation of TGF-β expression and the suppression of Smad signalling molecules. The methodology involved analysing data from various studies to understand ghrelin’s molecular mechanisms in combating fibrosis.

**Results:**

The findings from the reviewed studies indicate that ghrelin exerts significant anti-fibrotic effects across multiple organ systems. Specifically, ghrelin was found to downregulate TGF-β expression and suppress Smad signalling molecules, leading to a marked reduction in fibrous tissue accumulation and preservation of organ function. In liver fibrosis models, ghrelin reduced TGF-β1 levels and Smad3 phosphorylation, while in kidney and cardiac fibrosis, similar protective effects were observed. The data also suggest that ghrelin’s effects are mediated through both canonical and non-canonical TGF-β pathways.

**Conclusions:**

Ghrelin presents a promising therapeutic agent in the management of fibrosis due to its potent anti-inflammatory and anti-fibrotic actions. Its ability to modulate the TGF-β signalling pathway underscores a vital molecular mechanism through which ghrelin can mitigate fibrotic progression in various organs. Future research should focus on further elucidating ghrelin’s molecular interactions and potential clinical applications in fibrosis treatment, offering new avenues for developing effective anti-fibrotic therapies.

## Introduction

When severe necrosis or apoptosis occurs, fibrosis, an intrinsic response to chronic injury, helps preserve organ integrity. However, worsening injuries can lead to significant scarring or even organ failure (Ref. 1). Fibrosis, which affects various tissues, follows similar pathological mechanisms. During the inflammatory stage, macrophages release Transforming Growth Factor-beta (TGF-β), which induces extracellular matrix (ECM) protein production, transforms fibroblasts into myofibroblasts, and leads to tissue fibrosis (Ref. 2). TGF-β ligands are potent drivers of ECM deposition and have a natural affinity for the ECM, creating a concentrated pool of pro-fibrotic factors at injury sites, with TGF-β being a key cytokine. TGF-β alters intercellular connections, stimulates matrix protein synthesis, and inhibits matrix degradation. Interaction of TGF-β with its receptor activates fibroblasts into myofibroblasts, thereby promoting fibrosis (Ref. 3). Prolonged TGF-β production aggravates fibrosis, making TGF-β signalling a key target in treatment research. Fibrosis, which can be potentially fatal, can impair multiple organs, including the liver, kidneys, heart, lungs, and skin. Therefore, there is still a high demand for effective and safe therapeutic strategies targeting fibrogenesis.

Ghrelin, a gastric peptide and endogenous ligand for GHSR-1a, was first identified in the stomachs of rats (Ref. 4). It exists in acylated and desacyl forms; the acylated form is known as active ghrelin, while the desacyl form is non-active (Ref. 5). The acylated form, known for its high affinity for GHSR-1a, is biologically active and promotes growth hormone release, food intake, adiposity, and positive energy balance (Refs. 6, 7). Recent studies have revealed ghrelin’s anti-inflammatory and anti-fibrotic properties in various tissues. It suppresses TGF-β expression, aiding in the reduction of hepatic fibrosis (Ref. 8), and alleviates heart function in heart failure rats, reducing myocardial fibrosis (Ref. 9). Ghrelin also reduces pulmonary fibrosis by regulating inflammatory factors (Refs. 10, 11). Altered levels of ghrelin in fibrosis-related conditions suggest its role in tissue homeostasis or pathology, although its molecular antifibrotic mechanisms remain unclear (Table 1) (Refs. 12–21).

**Table 1.** Changes of ghrelin, and obestatin blood concentrations in human pathological conditions leading to organ fibrosis

Pathological condition	Ghrelin	Obestatin	Correlation	References
Liver cirrhosis	↓	nd	Ghrelin levels negatively correlate with cirrhosis severity	(12)
Primary biliary cirrhosis	↑	nd	Ghrelin levels positively correlate with cirrhosis severity	(13)
Cholestasis	↓	nd	Ghrelin levels negatively correlate with fibrosis severity	(14)
Hepatic Hydatidosis	↓	nd	Ghrelin levels negatively correlate with fibrosis severity	(15)
Chronic kidney disease (CKD)	↑	↓	ghrelin levels positively correlate with fibrosis severity	(16)
Chronic heart failure (CHF)	↓	nd	Ghrelin levels positively correlate with a favourable prognosis	(17)
Chronic obstructive pulmonary disease (COPD)	↑	nd	Ghrelin levels positively correlate with inflammation	(18)
Sepsis	↑	nd	ghrelin levels positively correlate with Sepsis severity	(19)
Cystic fibrosis	↑	nd	ghrelin levels positively correlate with fibrosis severity	(20)
Systemic sclerosis	↓	nd	Ghrelin levels negatively correlate with systemic sclerosis	(21)

This review provides the latest evidence for the anti-fibrotic effects and mechanisms of ghrelin and further elucidates its role in fibrotic diseases by regulating the TGF-β signalling pathway. Additionally, the review discusses the different regulatory modes of TGF-β signalling pathways and the potential of ghrelin receptor antagonists as effective therapeutic drugs for various diseases.

## Role of ghrelin in tissue fibrosis

### Characteristics of Ghrelin

**Figure 1.**
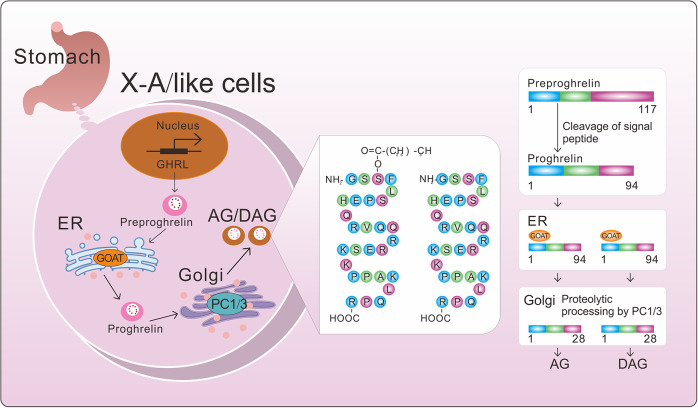
The acylation of Ghrelin. In X/A-like cells of the stomach, proghrelin is generated by specifically cleaving the signal peptide of preproghrelin, and then localized to the ER, where GOAT acylates proghrelin at Ser3 with n-octanoic acid. In the Golgi body, acylated proghrelin is transported so that PC 1/3 can cleave it and create a 28-amino acid AG, PC1/3 might also cleave non-acylated proghrelin to produce DAG. ER, endoplasmic reticulum; GOAT, ghrelin o-acyltransferase; PC1/3, prohormone convertase 1/3; AG, acylated ghrelin; DAG, desacyl ghrelin.

Many tissues and organs, including the peripheral and central nervous systems, express high levels of ghrelin and GHS-R1a. Organs that express ghrelin genes at various developmental stages in humans and rodents include the intestine, brain, heart, lungs, testicles, immune cells, and pancreas (Refs. 22, 23). However, ghrelin is primarily synthesized by gastric fundic gland X/A cells (Ref. 4) (Figure 1). Ghrelin is a 28-amino acid peptide, with the third amino acid initially unidentified. A study on rat ghrelin cDNA indicates that the third amino acid residue is serine, which undergoes acylation at Ser3 by Ghrelin O-acyltransferase (GOAT), a member of the membrane-bound O-acyltransferase family, using n-octanoic acid (Ref. 24). Ghrelin and GOAT have similar tissue expression profiles in humans and mice, with notable expression in the human pancreas and stomach, and in the mouse stomach and small intestine (Ref. 25). Acylated ghrelin, which binds to GHSR-1a, is considered biologically active. The 117-amino acid preproghrelin gene, which encodes ghrelin, is cleaved to produce 94-amino acid proghrelin. This cleavage and subsequent acylation by GOAT in gastric X/A-like cells occur in the endoplasmic reticulum (ER) (Ref. 26). Prohormone convertase 1/3 (PC1/3) then hydrolyzes acylated proghrelin in the Golgi, resulting in acylated ghrelin (AG) (Ref. 27). Desacyl ghrelin (DAG) is also produced when PC1/3 cleaves non-acylated ghrelin (Ref. 26) (Figure 1). Interestingly, desacyl ghrelin also exerts unique activities, regulating glucose and lipid metabolism. DAG consistently lowers mitochondrial ROS production, enhances insulin signalling, and promotes bone marrow adipogenesis (Refs. 28, 29). However, studies on receptors and regulatory pathways related to desacyl ghrelin are still lacking. Notably, acylated ghrelin exhibits antifibrotic properties, reducing tissue fibrosis by diminishing fibroblast activity, which is often linked to decreased inflammatory factors. It mitigates inflammation and fibrosis by inhibiting the nuclear factor kB (NF-kB) pathway (Ref. 30). Furthermore, ghrelin suppresses the expression of Smad3 and TGF-β1. Given TGF-β’s role as a pro-fibrotic cytokine and the limited effectiveness of pharmaceutical interventions targeting it, there is a pressing need for new pathway targets in the treatment of fibrosis.

### Antifibrotic effect of ghrelin in different organs

Ghrelin has been confirmed to improve ischemic injury, reduce inflammatory damage and fibrosis formation, accelerate tissue repair, and exert other protective effects in target organs such as the brain, heart, gastrointestinal tract, pancreas, and kidneys. Research indicates that ghrelin primarily regulates inflammatory factors to produce anti-fibrotic effects. Ghrelin has been reported to reduce lung damage by inhibiting the nuclear factor kB (NF-kB) pathway (Ref. 11). Clinical research suggests that boosting ghrelin expression in elderly patients with liver damage or chronic liver disease may help restore and regenerate the liver while modestly inducing autophagy (Refs. 31, 32). Ghrelin improves myocardial infarction by downregulating inflammatory cytokines such as IL-1β and TNF-α (Ref. 33). Furthermore, ghrelin exerts its antifibrotic effects by inhibiting the TGF-β/Smad3 pathway (Ref. 34). Ghrelin can also regulate immune homeostasis and fibrosis, but more research is needed to understand the interaction and crosstalk between these two processes.

## The role of TGF-β in fibrosis

### Mechanisms of TGF-β activation and signalling in fibrotic tissue response

**Figure 2.**
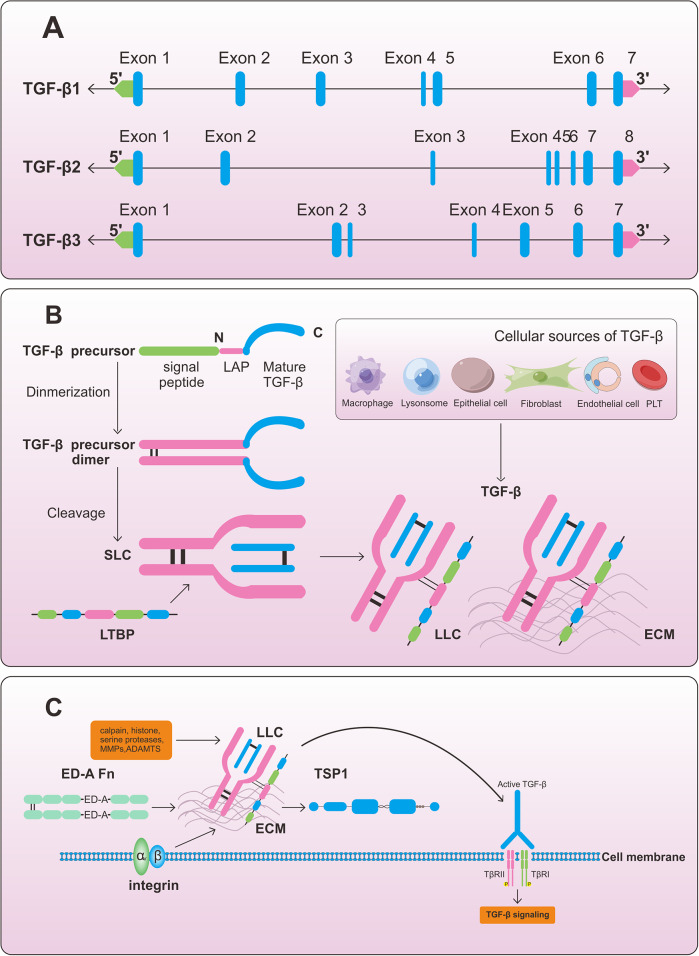
The gene TGFB(A) and TGF-β activation. A Gene structure of TGF-β1, TGF-β2, and TGF-β3: The untranslated regions of 5′ and 3′ are marked in green and pink, respectively, while exons are represented in blue. B TGF-β is produced by various cell types. With a signal peptide in the large terminal portion called the latency-associated peptide (LAP) and a C-terminal fragment for mature TGF-β, pro-TGF-β is synthesized as a latent complex in the ECM. With the removal of the signal peptide, the precursor protein is dimerised. Following proteolytic cleavage, the TGF-β dimer binds non-covalently to mature TGF-β to form SLC. Then, SLC generally binds to LTBP, forming LLC. The LLC is then secreted into ECM. C The release of mature TGF-β from the latent form involves protease, integrin (ITG), ED-A Fn and matrix protein-mediated actions. These active TGF-β ligands bind to the TGFβRI/TGFβRII receptor complex on the cell surface and initiate intracellular TGF-β signalling. ECM, extracellular matrix; SLC, small latent complex; LTBP, latent TGF-β binding proteins; LLC, large latent complex.

TGF-β plays a critical role as an effector in tissue fibrosis, being released by various cell types, including macrophages, lymphocytes, epithelial cells, fibroblasts, pericytes, endothelial cells, and platelets, in response to damage (Ref. 3) (Figure 2B). There are three isoforms of TGF-β (β1, β2, and β3), and the specific cellular source of TGF-β depends on the nature of the damage and the cellular composition of the affected organ (Figure 2A). TGF-β is primarily released and retained in the extracellular matrix (ECM) as a latent complex (Ref. 35). For TGF-β to carry out its biological functions, it must first be activated. This homodimeric pro-protein consists of a signal peptide in the large N-terminal portion, called the latency-associated peptide (LAP), and a C-terminal sequence that includes mature TGF-β monomers. Dimerization of the precursor protein occurs following the removal of the signal peptide (Ref. 36). Proteolytic cleavage then leaves the mature TGF-β dimer linked to the LAP prodomain, forming a small latent complex (SLC). The SLC combines with latent TGF-β binding proteins (LTBP) to create a large latent complex (LLC) (Ref. 37), which is then secreted into the ECM. Once released from the cell, TGF-β dimers bind to LTBP, transporting latent TGF-β to the ECM, where it remains inactive (Ref. 38) (Figure 2B).

Multiple mechanisms can activate TGF-β in fibrotic tissues. Proteases such as calpain, histone, serine proteases, matrix metalloproteinases (MMPs) (Ref. 39), and ADAMTS (a disintegrin and metalloproteinase with thrombospondin motifs), which can activate TGF-β in vitro and potentially in vivo, are linked to TGF-β activation in fibrotic lesions (Refs. 40–43) (Figure 2C). However, the relative importance of these proteases in TGF-β activation is unclear due to their complexity. Cell surface integrins (ITG) can also activate latent TGF-β, with αvβ6 integrins causing local TGF-β activation in fibrotic tissues either independently or protease-dependently. Inhibiting αvβ6 integrin expression can slow the progression of idiopathic pulmonary fibrosis by decreasing TGF-β activity (Ref. 44). Moreover, integrin binding to LAP and the action of MMP14 are essential for protease-mediated TGF-β activation. Promoting MMP14 proteasomal degradation can increase TGF-β expression (Ref. 45). Non-proteolytic activation might involve the actin cytoskeleton exerting tension on the LLC, leading to a conformational change that activates TGF-β and allows it to bind to its receptor (Refs. 46–48). Specialized matrix proteins such as fibronectin isoforms, fibulin-1c, and thrombospondin-1 (TSP-1) are also associated with TGF-β activation in fibrotic tissues. There is significant upregulation of ED-A Fn, a splice variant of fibronectin, in fibrotic tissues, which aids TGF-β activation by immobilizing LTBPs within the matrix and localizing activatable TGF-β to damaged areas (Ref. 49). Once activated, TGF-β ligands bind to TGF-βRI/TGF-βRII receptors on the cell surface, enabling them to participate in TGF-β signalling (Figure 2C).

TGF-β, essential in tissue fibrosis, acts on fibroblasts and directly impacts fibroblast-driven tissue fibrosis, as current research shows. This impact is evident in mouse models of fibrosis, where fibrotic responses significantly decrease following the specific deletion of TβRs from fibroblasts (Refs. 50, 51). TGF-β’s fibrotic effect primarily involves inducing phenotypic changes in myofibroblasts, marked by abundant vimentin-positive intermediate filaments (Ref. 52). Unlike fibroblasts, myofibroblasts have numerous actin-myosin bundles connected to the matrix, which may influence scar contraction. TGF-β also inhibits fibrosis by triggering protease inhibitors like tissue inhibitors of metalloproteinase (TIMP) and plasminogen activator inhibitor 1, protecting the matrix from degradation (Ref. 53). Prolonged TGF-β production in fibrotic diseases correlates with the severity of fibrotic changes, making TGF-β a key therapeutic target due to its diverse functions and roles in disease. However, there are significant challenges in clinical translation due to the complex biochemistry of TGF-β. Persistent suppression of TGF-β production could lead to unacceptable toxicity (Ref. 54). For example, patients with early diffuse cutaneous systemic sclerosis (SSc) treated with fresolimumab, a humanized TGF-β monoclonal antibody, experienced adverse reactions such as gastrointestinal bleeding, epistaxis, gingival bleeding, and anaemia (Ref. 55). Research also showed that selective knockout of TGF-βRII in renal tubular cells after ureteral obstruction increased renal inflammation and upregulated IL-1β and tumour necrosis factor-α (TNF-α) (Ref. 56). Currently, no known adverse effects are associated with long-term ghrelin therapy for fibrotic disorders. It is hoped that outcomes from preclinical animal studies will be replicated in human clinical trials.

### Comprehensive strategies for TGF-β inhibition in fibrotic disease treatment

**Figure 3.**
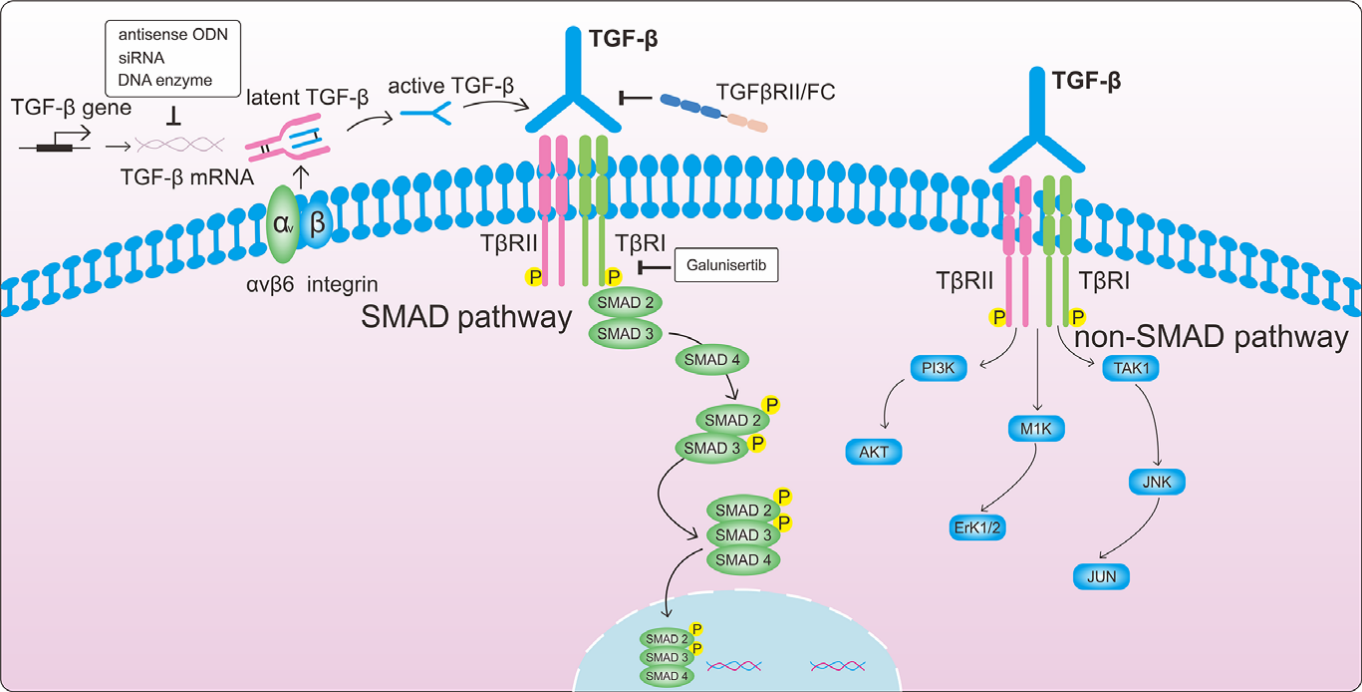
Targeting TGF-β signalling. Firstly, by inhibiting TGF-β from being translated and transcribed. Antisense oligodeoxynucleotides (ODN) (Trabedersen), siRNAs, or DNA enzymes can all inhibit the transcription or translation of TGF-β mRNA. Secondly, inhibition of latent TGF-β activation. Fibrosis can be treated with anti- αvβ6 integrin antibody while αvβ6 integrin activates TGF-β, and anti-αvβ6 antibody decreases collagen production and partially inhibits TGF-activity. Thirdly, Inhibition of activated TGF-β binding to its receptor by ligand trapping. TGFβRII/Fc, which consists of the extracellular domain of the TGFβ type II receptor combined with an immunoglobulin heavy-chain Fc fragment, efficiently hindered TGFβ1 to binding with the extracellular receptor. Fourthly, by blocking intracellular TGF-β signalling. Galunisertib is an oral inhibitor of TGF-β type I receptor kinase, and it inhibited the phosphorylation of Smad2/3, thereby blocking TGF-β signalling. TGF-β signalling is mediated via non-SMAD pathways, including PI3K/AKT, ERK and JNK, which can lead to ECM creation and myofibroblast activation. PI3K/AKT, phosphatidylinositol 3-kinase/protein kinase B; ERK(MAPK), mitogen-activated protein kinase; JNK, c-Jun N-terminal kinase. (The grey arrow means promotion and the T bar means inhibitory effect.).

TGF-β inhibition has been the principal therapeutic strategy used in human patients and animal trials for fibrotic diseases. Studies targeting TGF-β have been conducted in several contexts. Numerous studies have demonstrated that the following routes primarily regulate the expression of TGF-β at the protein level. Firstly, by inhibiting TGF-β translation and transcription. TGF-β-targeting anti-renal fibrosis gene therapy has been used clinically. It is necessary to inject genetic material into glomerulosclerotic mesangial cells or interstitial fibroblasts in individuals with renal fibrosis to inhibit TGF-β transcription and translation. Numerous methods, including antisense ODN, DNAzyme (Refs. 57, 58), and short interfering RNA (siRNA) (Ref. 59) (Figure 3), have been found to decrease TGF-β expression in the thylakoid and mesangial cells. Secondly, it inhibits latent TGF-β from being activated. The activation of TGF-β in pulmonary fibrosis is mostly dependent on αvβ6 integrin (Figure 3). Small doses of anti-αVβ6 antibody decreased collagen expression and partially blocked TGF-β activity in lung fibrosis-modelling mice without increasing alveolar inflammatory cell populations or activating macrophages. This may inhibit TGF-β at sites where αvβ6 integrin has upregulated it without affecting other homeostatic effects of TGF-β (Ref. 60). Thirdly, exploiting ligand traps that hinder activated TGF-β from attaching to its receptor TGFβR I and TGFβR II. Type II receptors on the cell surface bind to activated TGFβ, and these receptors recognize TGFβ1 ligands. While the monomeric, inactive soluble type II receptor may prevent TGF-β1 from acting as a ligand trap, its affinity for binding TGF-β1 is about ten times lower than that of the type II receptor on the cell surface (Ref. 61). In contrast to TGFβRII/FC, a chimeric protein consisting of the extracellular component of the TGF-βII receptor fused to the FC fragment of the immunoglobulin heavy chain, functions differently from the monomeric type II receptor in that it blocks TGF-β1 from binding to the cell surface receptor (Figure 3). TGFβRII/FC inhibited ECM production in a rat model of proliferative glomerulonephritis (Ref. 62). Fourthly, by inhibiting TGF-β signalling and its downstream signalling pathways. Activated TGF-β binds to receptors (TβRI/TβRII) to stimulate different downstream signalling pathways (SMAD and non-Smad pathways) to regulate context-dependent transcription (Ref. 63). TGF-β-induced activation of extracellular regulated protein kinases (ERK) and c-Jun N-terminal kinase (JNK) pathways results in Smad phosphorylation and affects Smad activation in non-Smad-dependent pathways (Ref. 64). Furthermore, TGF-β1 is activated and TGF-β responses are enhanced by TGF-β-induced Ras/extracellular signal-regulated kinase (ERK) MAPK signalling (Ref. 65). However, TGF-β mainly causes fibrosis via the Smad signalling pathway, whereby TGF-β type II receptor binding via the Smad-dependent route enables it to dimerize with the type I receptor and causes the type I receptor to become phosphorylated via the type II receptor kinase. Consequently, the activated type I receptor phosphorylates Smad2/3, which subsequently links forces with Smad4 to form a complex with Smad4. Target gene transcription is regulated by the active Smad complex, which translocates to the nucleus (Ref. 66). Smad2/3 phosphorylation is downregulated by galunisertib (LY2157299), an oral inhibitor of TGF-β type I receptor kinase. Galunisertib inhibited the phosphorylation of Smad2/3 but not Smad1 in a liver fibrosis model, which in turn reduced the maturation and synthesis of collagen (Ref. 67) (Figure 3).

Remarkably, a plethora of research conducted on animals has demonstrated that ghrelin reduces fibrosis mostly by obstructing TGF-β signalling in cells. For instance, ghrelin administration therapy dramatically decreased TGF-β1 and p-Smad3 protein expression in a mouse model of hepatic fibrosis, as well as reduced extracellular matrix formation and myofibroblast accumulation (Ref. 68). Ghrelin prevented postoperative adhesions in a mouse model of the disorder by downregulating pro-inflammatory factors TGF-β3 and TGF-βR2 and upregulating inhibitory proteins like Smad7 and Smad6. This reduced upstream collagen deposition and myofibroblast differentiation (Ref. 69).

## The role of Ghrelin in liver diseases through TGF-β signalling

### Therapeutic targets of TGF-β in liver fibrosis

Hepatic stellate cells (HSCs) play a crucial role in hepatic fibrosis, involving key proteins such as matrix metalloproteinases (MMPs) and tissue inhibitors of metalloproteinases (TIMPs). MMPs aid in ECM degradation, while TIMPs contribute to ECM preservation (Ref. 70). Activated HSCs produce myofibroblasts (MFBs), which are significant sources of collagen and ECM proteins (Ref. 71). Interestingly, HSCs may evolve into hepatocellular carcinoma (HCC) cells (Ref. 72). Abnormal TGF-β signalling in HSCs leads to pathological fibrosis and tumorigenesis, driven by excessive ECM deposition (Ref. 73). Following liver cell injury, TGF-β can inhibit AMPK/mTOR signalling pathways while activating TGF-β1/Smads signalling pathways, promoting autophagy and inducing HSC proliferation and activation (Ref. 74). This is evident in CCL4-induced liver fibrosis models, where increased expression of TGF-β1, MMP2, MMP9, and Smad2/3 genes is observed (Ref. 75). Additionally, HSCs release collagen triple helix repeat containing 1 (CTHRC1), which promotes TGF-β signalling and facilitates the transformation of HSCs from quiescence to activation (Ref. 76). CTHRC1 mAb treatment in hepatic fibrosis models reduced collagen deposition and TGF-β1 mRNA levels by inhibiting Smad2 and Smad3 phosphorylation, thereby blocking TGF-β signalling (Ref. 77).

**Figure 4.**
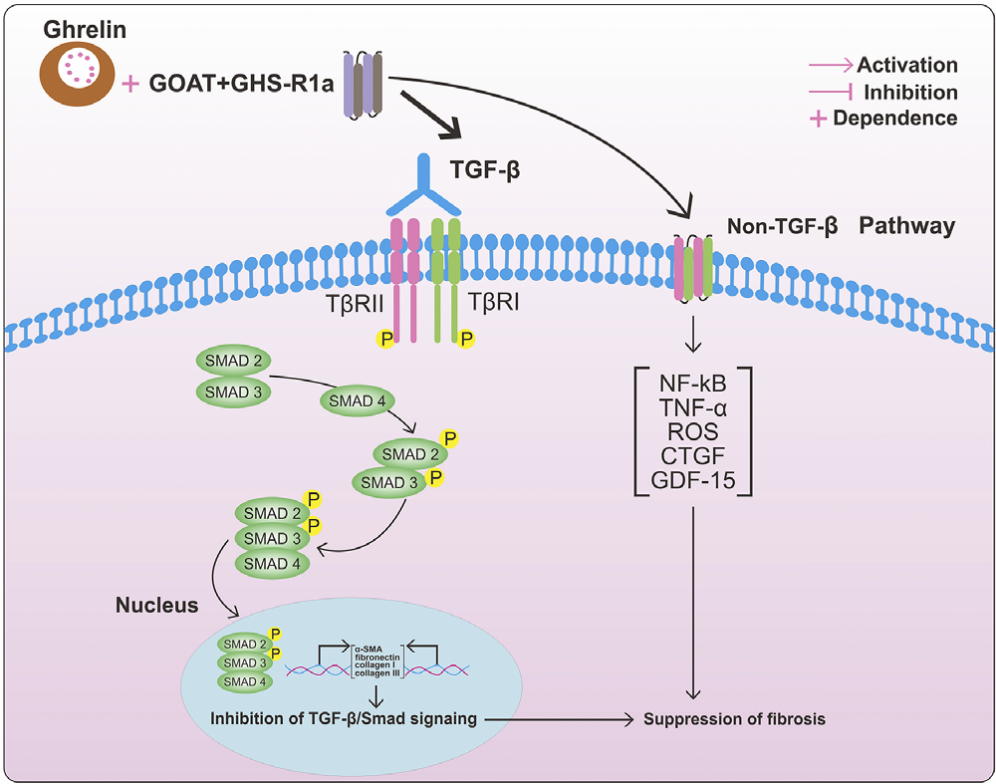
TGF-β signalling pathway and The roles of ghrelin in fibrosis. When TGF-β is activated, it phosphorylates TβR II, which recruits TβR I to phosphorylate the Smad protein receptor. Phosphorylated Smad 2/3 then combines with Smad4 to form a complex. Target gene transcription is regulated by the activated Smad complexes once they are transported to the nucleus. In renal fibrosis, exogenous ghrelin administration decreased TGF-β1, Smad, and p-Smad3 protein expression. Collagen I, collagen III, α-SMA, and fibronectin expression levels were all crease; however, after ghrelin treatment, they all decreased. Amelioration of fibrosis via the TGF-β1-Smad pathway. Ghrelin can also exert anti-fibrotic effects through non-TGF-β pathways, such as NF-kB, TNF-α, ROS, CTGF, and GDF-15. α-SMA, α-smooth muscle actin; NF-kB, Nuclear factor kB; TNF-α, Tumor necrosis factor-α; ROS, Reactive oxygen species; CTGF, Connective tissue growth factor; GDF-15, Growth differentiation factor 15. (The grey arrow means promotion and the T bar means inhibitory effect.).

Numerous studies have demonstrated the role of Smad proteins in mediating intracellular TGF-β signalling, with TGF-β1 being particularly significant due to its involvement in various pathological processes (Ref. 78). TGF-β1 activates both canonical and non-canonical pathways, including JNK, PI3K/AKT, and ERK, leading to ECM production and myofibroblast activation (Ref. 79) (Figure 3). The release of activated TGF-β1, binding to the type II TGF-β1 receptor (TβRII), initiates the TGF-β1/Smad signalling pathway. TβRII then recruits and phosphorylates TβRI, making TβRI more sensitive to its substrates Smad2 and Smad3. TβRI phosphorylates Smad2 and Smad3, creating pSmad2 and pSmad3, which then form a complex with Smad4. This phosphorylation results in the formation of complexes that enter the nucleus and affect gene transcription (Ref. 80) (Figure 4). Research indicates that deregulation of the Smad pathway plays a significant role in the pathophysiology of hepatic inflammation, fibrosis, and HCC (Ref. 81). In one study, hepatocyte migration and proliferation were reduced, and hepatic inflammation, fibrosis, and epithelial-mesenchymal transition were mitigated in Smad4 knockout mice. This also led to a reduction in DNA-binding protein inhibitor (ID1) expression and connective tissue growth factor (CTGF) secretion (Ref. 82). Furthermore, TGF-β-stimulated type I collagen expression in fibroblasts was modulated by alterations in Smad3 and Smad2 levels, suggesting that Smad2 is less effective than Smad3 in stimulating fibroblasts in response to TGF-β (Ref. 83). These findings emphasize the importance of targeting Smad signalling in preventing TGF-β activation.

Interestingly, for HSC activation to occur, cells must undergo metabolic reprogramming. Indeed, energy is essential for HSC activation. The primary metabolic change related to glucose involves a transition from aerobic glycolysis to oxidative phosphorylation (Ref. 84). A single glucose molecule in the tricarboxylic acid (TCA) cycle produces 32 adenosine triphosphate (ATP) molecules, whereas glycolysis produces only two ATP molecules. Although glycolysis is less efficient than oxidative phosphorylation in ATP production, its faster rate makes it more effective in generating the required ATP for HSC activation (Ref. 85). Researchers have noted that glycolysis produces precursors for biomass synthesis, including lipids, nucleotides, and amino acids, all essential for mitosis (Ref. 86). Given that TGF-β induces glycolysis, this signalling molecule may facilitate metabolic reprogramming. In idiopathic pulmonary fibrosis (IPF), a mechanistic study demonstrated that TGF-β leads to lactate accumulation through an induced glycolytic pathway, causing a decrease in microenvironmental pH. This, in turn, activates latent TGF-β in the stored ECM, creating a positive feedback loop that amplifies TGF-β activity (Ref. 87). Therefore, anti-hepatic fibrosis treatments might effectively target glycogen metabolism. Additionally, TGF-β signalling is involved in amino acid metabolism. Recent evidence shows that TGF-β-induced HSC transdifferentiation depends on glutamine metabolism (Ref. 88). Glutamine catabolism occurs in two steps: first, glutaminase (GLS1) converts glutamine to glutamate; second, glutamate dehydrogenase or transaminase converts glutamate to α-ketoglutarate, which is subsequently incorporated into the TCA cycle by glutamine metabolites (Ref. 89). TGF-β increases GLS1 in activated HSCs through the p38 MAPK and Smad3 pathways. Conversely, depleting extracellular glutamine or silencing GLS1 in the presence of glutamine inhibits TGF-β-induced HSC activation and reduces the expression of pro-fibrotic markers (Refs. 89, 90). These findings suggest that TGF-β is also involved in the progression of metabolic liver disease.

These findings clearly indicate that TGF-β signalling promotes HSC metabolism, thereby enhancing liver fibrosis. Although fibrosis can be reversed, it often continues to progress. A recent study revealed that ghrelin reduced hepatocyte death in chronic cholestasis and reversed early pathophysiological changes associated with hepatic fibrosis (Ref. 14). Thus, blocking TGF-β-induced HSC activation could reverse early liver tissue injury.

### Ghrelin and liver diseases

There is a close and complex regulatory relationship between ghrelin and the liver. Ghrelin-deficient mice are more susceptible to acute and chronic liver injury than normal mice (Ref. 8); however, ghrelin treatment or GHS-R agonist administration decreases the extent of acute or chronic liver fibrosis. Consequently, ghrelin may have a beneficial effect on hepatic fibrosis. The reduction in pro-inflammatory factor activation is linked to ghrelin’s anti-fibrotic effects. In the bile duct ligation (BDL) rat model of hepatic fibrosis, hepatic malondialdehyde (MDA) levels, myeloperoxidase (MPO) activity, and collagen deposition were all higher in the BDL group. Serum aspartate transaminase (AST), alanine aminotransferase (ALT), and lactate dehydrogenase (LDH) levels, as well as plasma levels of tumour necrosis factor-α (TNF-α), interleukin-1β (IL-1β), and interleukin-6 (IL-6), were also higher in the BDL group but significantly lower in the ghrelin-treated group (Ref. 8). Thus, it is speculated that exogenously administered ghrelin reduces the severity of hepatic fibrosis caused by biliary obstruction by inhibiting the expression of pro-inflammatory factors (Ref. 91). Additionally, a study on acetaminophen-induced hepatotoxicity revealed that acetaminophen dramatically raised liver ALT/AST levels, caused hepatocellular damage, and considerably increased TNF-α levels. However, ghrelin administration may partly mediate its hepatoprotective effects through an anti-inflammatory mechanism (Ref. 92). Obesity plays an important role in the development of nonalcoholic steatohepatitis (NASH). It has been reported that ghrelin inhibits the progression of lipopolysaccharide (LPS)-induced NASH by decreasing M1 polarization of Kupffer cells mediated by GHSR1a, reducing levels of TNF-α and inducible nitric oxide synthase, and increasing Arg1 (Ref. 93). The study revealed the interaction between gut-derived LPS and the gastric endocrine hormone ghrelin. In a rodent model of cholestasis, serum ghrelin levels are reduced. Ghrelin administration activates the AMPK pathway through GHS-R1a binding, reducing cholangiocyte proliferation and liver fibrosis markers (TGF-β, PDGF-α, CTGF, MMP2, MMP-1). The results indicated that ghrelin plays an antifibrotic role in the context of cholestasis (Ref. 14). Additionally, serum ghrelin levels can be used as an indicator for monitoring liver cirrhosis.

Remarkably, ghrelin reduces systemic inflammation through its sympathoinhibitory actions (Ref. 94). The rostral ventrolateral medulla (RVLM), a significant source of sympathoexcitatory signals to the periphery, receives excitatory inputs from the paraventricular nucleus (PVN) and inhibitory inputs from the caudal ventrolateral medulla (CVLM), the nucleus tractus solitarius (NTS), and the nucleus ambiguous (NA). Ghrelin inhibits sympathetic output by blocking excitatory input from the PVN to the RVLM (Ref. 26). In patients with sepsis, centrally or peripherally administered ghrelin lowers circulating levels of TNF-α and norepinephrine (NE) (Ref. 95). Tissue damage leads to NE release from postganglionic sympathetic neurons, activating the α2A-adrenergic receptor (α2A-AR), which triggers MAPK p38 activation and TNF-α production in Kupffer cells, while ghrelin attenuates sepsis by reducing sympathetic nerve activity (Ref. 26). Ghrelin has been observed to activate the vagus nerve in septic rats, significantly lowering TNF-α and IL-6 levels and downregulating pro-inflammatory cytokines (Ref. 96), suggesting that its anti-inflammatory actions are mediated via ghrelin receptors on the vagus nerve. The exact mechanism by which ghrelin prevents hepatic fibrosis through the vagal pathway remains unclear.

### Ghrelin attenuates hepatic fibrosis through TGF-β Signalling

While ghrelin’s anti-inflammatory properties aid in protecting against liver injury, TGF-β signalling is the primary biochemical mechanism by which ghrelin prevents liver fibrosis. It is widely believed that ghrelin reduces hepatic fibrosis via the traditional Smad-dependent TGF-β pathway, including hepatic fibrosis induced by CCL4 in a rodent model. In this model, the CCL4-induced hepatic fibrosis group showed significantly higher levels of TGF-β1, Smad3, and p-Smad3, whereas the CCL4 + ghrelin-treated group exhibited significantly lower levels of these markers, suggesting that ghrelin may reduce hepatic fibrosis by inhibiting Smad phosphorylation and blocking intracellular TGF-β signalling (Ref. 68). Moreover, non-canonical Smad-independent TGF-β pathways also decrease liver fibrosis. An experiment on non-alcoholic fatty liver disease (NAFLD) demonstrated that ghrelin prevents liver injury by reducing ALT/AST levels, oxidative stress, inflammation, and apoptosis while restoring hepatic lipid metabolism both during and after NAFLD induction. The study found that NAFLD significantly reduced the phosphorylated forms of LKB1, AMPK, Akt, and PI3K, but not their total levels. Ghrelin administration restored the phosphorylation levels of these kinases to control levels without affecting their total levels, possibly mediated by targeting the LKB1/AMPK and PI3K/Akt pathways (Ref. 97). However, the interaction between these pathways and the canonical Smad-dependent pathways is not fully understood.

The role of ghrelin in liver function is dichotomous, as evidenced by earlier research. On the one hand, the expression of two indicators of liver fibrosis, collagen I and TGF-β1, was decreased in both mice and in vitro when ghrelin was administered to unstimulated HSCs. Conversely, ghrelin has no inhibitory effect on pro-inflammatory factors in vitro but enhances the activation of pro-inflammatory cytokines in vivo. A prior study discovered a significant correlation between the expression of genes associated with fibro cytokines and ghrelin levels in liver plasma (Ref. 8). Furthermore, compared to patients with mild fibrosis, patients with alcoholic hepatitis and chronic hepatitis C had significantly lower ghrelin levels in peripheral blood. These findings suggest that ghrelin is a novel prognostic indicator for patients with liver fibrosis and a marker of the severity of chronic hepatitis (Ref. 8). These findings imply that the TGF-β pathway is involved in the role of ghrelin in the progression of hepatic fibrosis.

## The role of ghrelin in renal disease through TGF-β signalling

### Therapeutic targets of TGF-β in renal fibrosis

Renal fibrosis, often leading to end-stage renal failure, is a complex process associated with the progression of many kidney diseases. However, its aetiology remains not fully understood. The healing of damaged renal tissue occurs in two stages: (1) the regenerative stage, where damaged cells are replaced by similar cells, and (2) the fibrotic stage, where connective tissue formation occurs. In renal parenchymal disorders, such as pyelonephritis and renal blood supply defects, fibrosis plays a protective role (Ref. 98). Under stressful conditions, fibrosis can progress to scarring, helping to maintain kidney structure integrity. However, if uncontrolled, excessive fibrosis may lead to pathological fibrosis, despite the initial benefits of the fibrotic phase. Pathological fibrosis is characterized by extracellular matrix deformation, leading to a serious, recurring, and chronic condition (Ref. 99).

TGF-β is abundantly expressed in renal fibrosis and plays a vital role in its onset and progression. Among the isoforms, TGF-β1 is the most significant pro-fibrotic factor in renal disease (Ref. 100). Renal fibrosis is significantly reduced when TGF-β1 is blocked, either by neutralizing TGF-β antibodies or antisense oligonucleotides, confirming TGF-β1’s pro-fibrotic effect (Refs. 101, 102). Numerous experimental studies have focused on targeting TGF-β for treating renal fibrosis. Klotho, an anti-ageing protein, is extensively expressed in renal tubular epithelial cells, but its expression markedly decreases with age or renal injury (Ref. 103). Overexpression or exogenous supplementation of Klotho attenuates renal fibrosis and prevents kidney damage in various chronic kidney disease (CKD) models (Ref. 104). KP1, a peptide derived from the human Klotho protein, has been studied for its effects on TGF-β and downstream signalling pathways. KP1 inhibits TGF-β signalling, ameliorating renal fibrosis and restoring endogenous Klotho expression. Additionally, KP1 decreased the expression of TGF-β, Smad3, and p-Smad3(Ref. 105). KP1 may modulate TGF-β signalling blockage, although the mechanism by which it prevents TGF-β from binding to its receptor remains unclear. However, developing effective therapeutics against TGF-β is challenging due to its broad spectrum of functions in both normal physiology and disease. Complete inhibition of TGF-β may lead to adverse effects, such as the loss of its anti-inflammatory functions. For instance, patients with diabetic nephropathy who received neutralizing anti-TGF-β1 antibodies did not experience a reduction in disease progression (Ref. 106). Therefore, researchers are exploring more efficient treatment methods to address renal fibrosis. Partial inhibition of TGF-β-induced renal fibrosis without impairing its other effects is currently under investigation. Notably, indirect prevention of fibrosis through modulation of TGF-β signalling has been demonstrated. A recent study showed that TGF-β-stimulated human renal tubular epithelial cells express TGF-β/Smad3-interacting long non-coding RNA (lnc-TSI), which helps mitigate renal fibrosis (Ref. 107).

### Ghrelin attenuates renal fibrosis via TGF-β signalling

Several studies have demonstrated that ghrelin provides renal protection. Ghrelin mitigated acute kidney injury resulting from ischemia and reperfusion, with these benefits being mediated by the growth hormone-insulin-like growth factor 1 pathway (Ref. 108). In another study, ghrelin administration alleviated sepsis-induced acute kidney impairment by inhibiting pro-inflammatory cytokines (TNF-α, IL-1β, and IL-6) (Ref. 109). Ghrelin also offers protection against renal fibrosis. In a mouse model of angiotensin II (AngII)-induced renal injury, AngII increased blood pressure and decreased appetite, body weight, blood urea nitrogen (BUN), urine protein, neutrophil gelatinase-associated lipocalin (NGAL), and neutral alpha-glucosidase (NAG) levels; however, daily intraperitoneal administration of ghrelin protected against AngII-induced renal injury. Ghrelin reduced senescent alterations and reactive oxygen species (ROS) elevations induced by AngII. AngII stimulated the ROS-producing enzyme NADPH oxidase (NOX), increasing expression of its NOX1 and NOX4 isoforms, while ghrelin down-regulated these molecules. AngII also increased expression of the NOX component p22phox, which was down-regulated by ghrelin treatment. Furthermore, AngII reduced the expression of peroxisome proliferator-activated receptor-c coactivator 1a (PGC-1a), a crucial regulator of mitochondrial biogenesis, which was counteracted by ghrelin. Ghrelin increased both mitochondrial biogenesis and UCP2 (mitochondrial uncoupling protein) expression. These findings suggest that ghrelin protects renal tissue by reducing oxidative stress, maintaining mitochondrial integrity, and promoting the expression of PGC-1a and UCP2. Additionally, ghrelin inhibited AngII-induced elevation of TGF-β and plasminogen activator inhibitor-1 (PAI-1), thereby improving renal fibrosis. The effects of ghrelin on AngII-induced kidney damage were found to be GHSR-dependent, as evidenced by increased renal tubular damage, renal dysfunction, oxidative stress, fibrosis, and senescence observed in GHSR-deficient animals. (Ref. 110). However, it remains unclear whether ghrelin’s renoprotective effect is mediated through comprehensive blockade of TGF-β signalling or specifically through blockade of the canonical Smad-dependent pathway downstream of TGF-β. In the unilateral ureteral obstruction (UUO) rat model, ghrelin treatment significantly decreased the expression of collagen I, collagen III, and fibronectin in the renal cortex. Ghrelin prevented the reduction of E-calmodulin and significantly decreased the expression of fibronectin and α-SMA, suggesting a reduction in fibroblast activation and epithelial-mesenchymal transition (EMT) in the obstructed kidney (Figure 4). Immunohistochemical staining and protein blotting analysis have shown that exogenous administration of ghrelin significantly reduced the expression of TGF-β1, Smad, and p-Smad3 proteins induced by UUO, indicating that ghrelin might inhibit TGF-β1/Smad3 signalling as part of its antifibrotic mechanism (Ref. 111) (Figure 4). By specifically blocking canonical Smad3 signalling, ghrelin could potentially reduce fibrosis without altering the other growth-dependent effects of TGF-β, thereby avoiding the negative effects associated with comprehensive TGF-β signalling blockade.

Studies have shown that the elevation of acyl-ghrelin (AG) in individuals with cystic fibrosis (CF) may be related to AG-mediated inhibition of insulin secretion. Sun et al. hypothesized that the elevation of AG helps reduce insulin secretion and hyperglycemia in CF ferrets. Fasting AG levels were increased in CF ferrets compared to non-CF ferrets. Interestingly, administration of the acyl-ghrelin receptor antagonist [D-Lys3]-GHRP-6 in non-CF ferrets impaired glucose tolerance. Insulin, glucagon-like peptide-1 (GLP-1), and gastric inhibitory polypeptide (GIP) responses were also eliminated during the glucose tolerance test. In contrast, [D-Lys3]-GHRP-6 improved glucose tolerance and enhanced the insulin-to-glucose ratio in CF ferrets but did not impact the already low GLP-1 and GIP levels. These results suggest that AG promotes glucose levels in CF and that reducing AG may improve hyperglycemia in cystic fibrosis. However, the mechanism underlying acyl-ghrelin elevation in CF has not been clarified in this report, and there is a lack of validation through in vitro experiments (Ref. 112).

Additionally, in the DOX-induced renal injury model, DOX can cause interstitial tubule fibrosis and cell proliferation, and upregulate the expression of SIRT1, TGF-β1, α-SMA, type I collagen, and TNF-α. However, acylated ghrelin downregulates the protein levels of TGF-β1, Smad3, and α-SMA through a SIRT1- and GHSR1β-dependent mechanism. To further demonstrate the regulatory mechanism of ghrelin, all these effects can be eliminated by using the antagonist [D-Lys3]-GHRP-6 (Ref. 113). Rikkunshito (RKT), a traditional Japanese medicine used to treat anorexia, has recently been found to play anti-fibrotic and anti-inflammatory roles through the ghrelin signalling pathway. RKT inhibits AngII-induced changes in the expression of genes associated with renal fibrosis and inflammation, such as type III collagen, TGF-β, monocyte chemotactic protein-1, and IL-6. RKT also restored the reduced expression of sirtuin 1, a key downstream pathway for ghrelin receptors. However, the details of the mechanism mediating ghrelin’s effects and whether blocking ghrelin receptors would eliminate RKT’s role remain unclear (Ref. 114). This focus on a single element of TGF-β signalling as an antifibrotic strategy could prevent the negative effects associated with comprehensive TGF-β signalling blockade.

## The role of ghrelin in cardiac disease through TGF-β signalling

### Therapeutic targets of TGF-β in cardiac fibrosis

Cardiac fibrosis, characterized by the replacement of normal myocardium with non-functional fibrotic tissue, ultimately leads to heart failure (Ref. 115). This process is primarily mediated by tissue-resident cardiac fibroblasts, which transform into myofibroblasts that secrete extracellular matrix (ECM) proteins, resulting in excessive ECM deposition. However, studies indicate that excessive ECM deposition is reversible, and even a modest reduction in collagen volume fraction (CVF), a critical indicator of ECM content, can improve cardiac function and coronary flow (Refs. 116, 117).

TGF-β is essential for activating fibroblasts and promoting ECM production in cardiac tissue, with TGF-β1 phosphorylating Smad3, leading to the development of cardiac fibrosis (Ref. 50). Targeting the TGF-β/Smad3 signalling pathway is a potential therapeutic approach for myocardial fibrosis. Research using a mouse model involving the selective deletion of Tgfbr1/2, Smad2, or Smad3 in fibroblasts revealed that while deleting Tgfbr1/2 reduced fibrosis, deleting Smad2 did not significantly alter the fibrotic response. However, deleting Smad3 markedly decreased fibrosis, suggesting that Smad3 is more efficient than Smad2 in activating fibroblasts during cardiac fibrosis (Ref. 50).

Therapeutic strategies to combat cardiac fibrosis include blocking TGF-β from binding to its receptor or inhibiting its intracellular signalling. Research on the use of Qishen Granule (QSG) for treating cardiac fibrosis has shown its effect on TGF-β pathway mediators, specifically Smad3 and glycogen synthase kinase-3β (GSK-3β), thereby reducing the severity of cardiac fibrosis (Refs. 118, 119). Additionally, SIRT1, a deacetylase, has been identified as having antifibrotic functions by regulating EndMT through the TGF-β/Smad2/3 pathway, which contributes to the attenuation of cardiac fibrosis (Ref. 120). This indicates that targeting downstream effectors of TGF-β signalling could offer new therapeutic opportunities.

### Ghrelin attenuates cardiac fibrosis via TGF-β signalling

Ghrelin has a protective effect on the cardiovascular system and may prevent heart failure and cachexia through various mechanisms, including improving energy metabolism and cardiac function (Ref. 9). Peripheral administration of ghrelin has been shown to enhance cardiac function and exercise capacity in individuals with chronic heart failure by increasing cardiac and skeletal muscle mass (Ref. 121). A very small dose of ghrelin injected into the nucleus accumbens of the medulla oblongata in rats decreased blood pressure, heart rate, and renal sympathetic nerve activity (Ref. 122). Similarly, subcutaneous (sc) injection of ghrelin in rats attenuated left ventricular (LV) remodelling after myocardial infarction (MI) by inhibiting cardiac sympathetic nerve activity (Ref. 123). These results suggest that ghrelin acts on the sympathetic nervous system to improve heart function. The anti-apoptotic function of active ghrelin was also observed in experimental MI animal models, where it activated the Raf-MEK1/2-ERK1/2 pathway and inhibited oxidative stress and inflammation through TLR4/NLR3 signalling (Ref. 124). In cardiac hypertrophy animal models, ghrelin may promote autophagy through the CaMKK/AMPK signalling pathway, exerting its protective effect on myocardial hypertrophy (Ref. 125).

While myocardial fibrosis results from various mechanisms, the exact process by which ghrelin inhibits myocardial fibrosis remains unclear. In one study, ghrelin stimulation of H9C2 cells (derived from rat BDIX heart myoblasts) promoted the expression of IL-33 and upregulated MEF2A and sST2. This regulation, which is EPAC-dependent, played a role in reducing myocardial apoptosis and promoting myocardial cell survival. In contrast, preliminary data on leptin stimulation of H9C2 cells showed the opposite effect of ghrelin treatment, suggesting that the balance of these two hormones affects IL-33 signalling. Obesity promotes IL-33 signalling and improves mild inflammation while increasing cardiovascular risk, a phenomenon that could be referred to as the “obesity paradox.” The role of ghrelin in cardiac remodelling and its association with the IL-33/ST2 pathway has been proposed, but further experiments involving ghrelin inhibitors are needed to fully investigate its role (Ref. 126).

A significant study demonstrated that TNF-α is downregulated during cardiomyocyte apoptosis and that ghrelin effectively reduces oxidative stress damage and doxorubicin-induced apoptosis in cardiomyocytes. TNF-α not only activates NF-κB, an anti-apoptotic factor, but also induces the expression of several anti-apoptotic genes such as c-FLIP, cIAP1, cIAP2, A1, A20, RAF1, TRAF2, and MnSOD. Conversely, ghrelin not only increases TNF-α gene expression but also enhances doxorubicin-induced NF-κB nuclear translocation (Ref. 127). In the pathological state of myocardial fibrosis induced by isoproterenol (ISO), the production of growth differentiation factor 15 (GDF15) was decreased, while the mRNA levels of type I and type III collagen were increased. The phosphorylation of Akt at Ser473 and GSK-3β at Ser9 was decreased with ISO, but ghrelin administration increased the levels of GDF15 mRNA and protein, significantly reversing the downregulation of p-Akt and p-GSK-3β (Ref. 128). It has been shown that ghrelin improves myocardial fibrosis through GDF15, which is expected to be an effective target for the treatment of myocardial fibrosis.

Myocardial fibrosis is significantly influenced by the cytokine TGF-β, which ghrelin downregulates to mitigate the disease’s effects. In an animal model of angiotensin II-induced cardiac fibrosis, the administration of exogenous ghrelin counteracted the substantial production of TGF-β1 and its downstream proteins, p-Smad2 and p-Smad3, effectively reducing the severity of the condition (Ref. 34). Additionally, another study revealed that desacyl ghrelin, through a GHSR-independent mechanism, decreased apoptosis and cardiac fibrosis. This form of ghrelin augmented the production of the anti-apoptotic protein Bcl-2, lowered the activity of cysteine protease-3 (caspase-3), and diminished doxorubicin-induced cardiomyocyte apoptosis and DNA damage (Ref. 129).

## The role of ghrelin in lung fibrosis

Studies have demonstrated ghrelin’s potent anti-inflammatory properties and its ability to curb the production and release of pro-inflammatory cytokines. By suppressing these factors, ghrelin safeguards against lung tissue injury (Ref. 130). Research on ghrelin’s pharmacological impact on lung injury shows that pretreatment with ghrelin protects alveolar epithelial cells from bleomycin-induced acute lung injury and reduces the expression of pro-inflammatory factors such as IL-1β and IGF-1. Ghrelin also preserves the integrity of alveolar epithelial cells by inhibiting fibroblast proliferation and stromal deposition, potentially reducing the fibrotic effects of IL-1β and IGF-1 (Ref. 131). Ghrelin has been shown to have a protective effect on acute lung injury induced by traumatic brain injury. Ghrelin administration improves traumatic brain injury-induced acute lung injury by blocking the NF-κB signalling pathway in a mouse model of brain trauma, although in vitro validation is lacking (Ref. 11). This suggests a promising treatment method for acute lung inflammation. In lung injury scenarios, TGF-β acts as a dual agent. Initially, it serves as an anti-inflammatory agent (Ref. 131), but as lung injury progresses, increased TGF-β levels promote lung fibrosis (Ref. 132).In addition, ghrelin alleviates pulmonary fibrosis through TGF-β, which may involve miR-125a-5p/ Ruppel-like factor 13 axis (Ref. 133). However, the exact role of ghrelin in suppressing fibrosis via TGF-β signalling remains unclear, indicating a need for further research on ghrelin’s anti-lung fibrosis mechanism, particularly focusing on the TGF-β pathway.

## Potential of ghrelin in other diseases

Researchers have confirmed that ghrelin exhibits anti-fibrotic effects across various fibrotic disorders. A notable study found significantly lower plasma levels of acylated and desacyl ghrelin in patients with systemic sclerosis (SSc). This indicates that ghrelin directly inhibits TGF-β1 expression and decreases collagen synthesis in dermal fibroblasts at the mRNA level (Ref. 21). By inhibiting TGF-β1, ghrelin may effectively control skin fibrosis. In a separate study, Hayan Jeong et al. uncovered the mechanism of ghrelin’s action in atopic dermatitis (AD). Ghrelin activates the glucocorticoid receptor (GR), which then collaborates with histone deacetylase 3 (HDAC3) and nuclear receptor corepressor (NCoR) to suppress the production of the thymic stromal lymphopoietin (TSLP) gene by binding to the negative glucocorticoid response element (nGRE) on its promoter. Additionally, ghrelin promotes the phosphorylation and inactivation of p300, causing protein kinase C (PKCδ) to localize to the nucleus. This reduces the acetylation and DNA binding activity of NF-κB p65 at the TSLP gene promoter. Through GR and PKCδ-p300-NF-κB-mediated regulation, ghrelin may reduce skin inflammation. The study highlights ghrelin’s crucial role in AD and provides new insights for AD research and treatment (Ref. 134). Ghrelin also exerts immunosuppressive effects. Subcutaneous injections of ghrelin and growth hormone (GH) in aged septic rats significantly lowered serum TGF-β levels. Surprisingly, vagotomy diminished the beneficial effects of GH and ghrelin treatments in these rats, suggesting GH and ghrelin reduce immunosuppression in aged sepsis rats through a vagal-dependent mechanism (Ref. 135). Additionally, cachexia, characterized by skeletal muscle atrophy, weight loss, and persistent appetite loss, benefits from ghrelin administration, especially in patients undergoing complete gastrectomy suffering from weight loss and appetite loss (Ref. 136). Ghrelin has been shown to have pro-appetite, energy metabolism, anti-inflammatory, anti-fibrotic, and cytoprotective effects via GHSR-dependent pathways (Refs. 137–140). The role of ghrelin receptor antagonists in modulating ghrelin’s effects remains a topic for further investigation.

## Ghrelin receptor antagonists

Ghrelin plays a key role in GHSR-1a activation, a widely distributed ghrelin receptor in both the central and peripheral nervous systems, is involved in several physiological actions of ghrelin. Regulating GHSR-1 signalling could benefit the treatment of conditions such as anorexia, cachexia, sarcopenia, metabolic disorders, neurological and neurodegenerative diseases, pain, and substance use disorders (Ref. 141). Ghrelin receptor antagonists with diverse molecular frameworks are emerging as potential therapeutic agents for a range of clinical disorders.

A recent discovery identified liver-expressed antimicrobial peptide 2 (LEAP-2) as an endogenous blocker of GHSR-1a, despite its initial classification as an antimicrobial peptide (Ref. 142). LEAP-2 inhibits ghrelin binding to GHSR, blocks ghrelin-mediated GH release and food intake, and helps maintain active glucose levels during chronic caloric restriction. The relationship between alterations in dietary status and circulating levels of ghrelin and LEAP-2 is inverse. Following a fast, serum LEAP-2 levels decreased while serum ghrelin levels increased. However, following a meal, serum LEAP-2 levels increased while serum ghrelin levels decreased, and mice with high LEAP-2 expression levels had lower ghrelin levels. Theoretically, this suggests that LEAP-2 may also prevent growth hormone-releasing peptides from being produced or secreted (Ref. 143). The study provides new perspectives for further research into disorders related to metabolism and obesity. A recent report that a long-acting LEAP-2 analog (LA-LEAP 2) can improve liver fat accumulation and inflammation provides a significant basis for further research and development of LEAP-2 analogs as potential drugs for the treatment of liver fat accumulation and related metabolic diseases (Ref. 144).

A study suggests that elevated serum ghrelin levels due to alcohol consumption impair pancreatic β-cell insulin secretion. The subsequent drop in serum insulin levels promotes the release of free fatty acids from adipose tissue, leading to hepatic steatosis. The primary cause is ghrelin’s inhibition of pancreatic β-cells’ ability to secrete insulin by binding to its receptor GHSR-1a and blocking Ca2+ inward flow. Another GHSR-1a antagonist, [D-Lys-3] GHRP-6, administered via peritoneal injections in alcohol-intake rats, improves insulin secretion, normalizes serum insulin levels, inhibits lipolysis, and reduces the liver’s absorption and synthesis of fatty acids, all of which lower the risk of alcoholic hepatic steatosis (Ref. 145).

**Figure 5.**
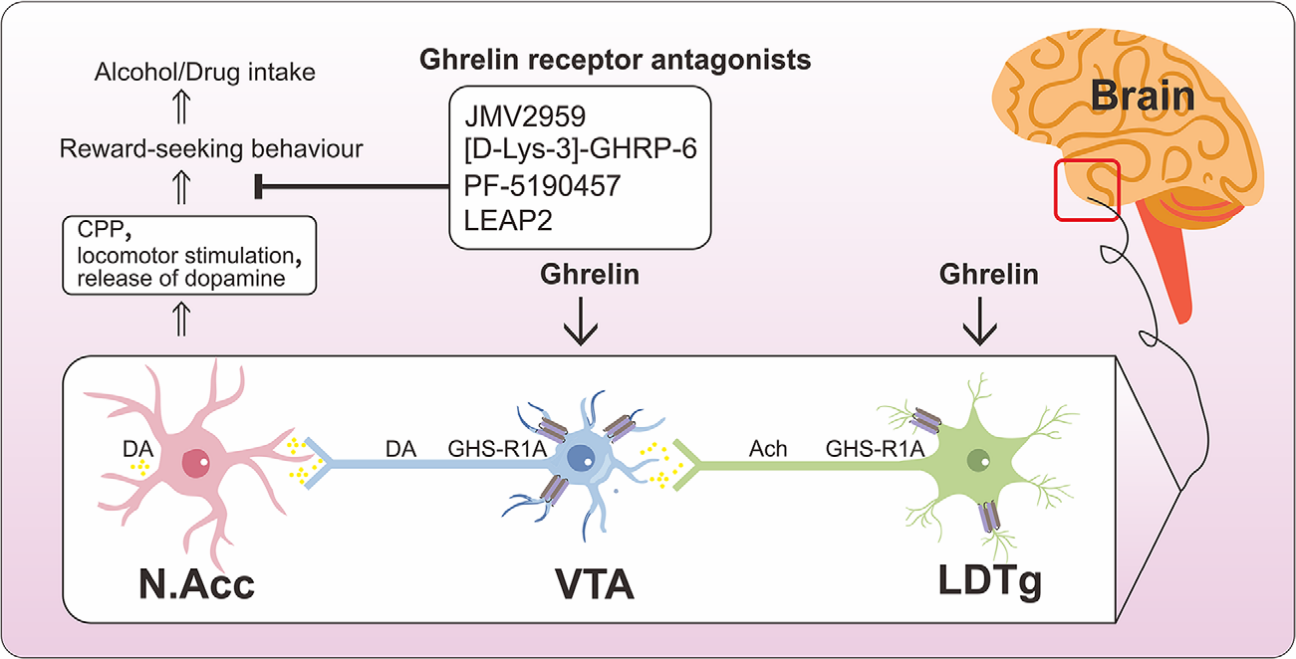
Ghrelin’s reward-seeking behavioural pathway. Ghrelin takes pre- and/or post-synaptic GHS-R1A signalling from VTA and LDTg to activate the cholinergic-dopaminergic reward link. This route increases DA release and activates VTA dopamine neurons that project to N.Acc, which stimulates reward-seeking behaviour. Ghrelin receptor antagonists reduce addictive drug-induced locomotor stimulation, dopamine release, and CPP. ACh, acetylcholine; DA, dopamine; LDTg, laterodorsal tegmental area; VTA, ventral tegmental area; N.Acc, nucleus accumbens, GHS-R1A, growth hormone secretagogue receptor; CPP, conditioned position preference.

Increased endogenous ghrelin levels caused by alcohol lead to lipid accumulation in hepatocytes, leaving the liver susceptible to inflammatory mediators and toxins, potentially aggravating the condition and leading to fibrosis (Ref. 145). Conversely, exogenous ghrelin injection alleviates liver fibrosis (Ref. 68). Thus, ghrelin has a dual role in liver tissue. It has been discovered that ghrelin plays a role in reward pathways triggered by alcohol or drug abuse (cocaine, nicotine, amphetamine). These pathways involve cholinergic inputs, primarily from the laterodorsal tegmental area (LDTg), and dopamine projections from the ventral tegmental area (VTA) to the nucleus accumbens (NAcc). Ghrelin enters the VTA or the LDTg to initiate a “cholinergic-dopaminergic” reward link. Ghrelin activates the mesolimbic dopamine system by binding to GHSR-1a in the VTA and LDTg (Ref. 146). Intravenous or central administration of ghrelin increases dopamine release in the accumbal area, causing an increase in NAcc dopamine turnover, conditioned place preference (CPP), and locomotor stimulation (Ref. 147). Ghrelin antagonists have thus been suggested as a potential new approach to treating addictive behaviours. JMV2959, a GHSR-1a antagonist, was shown to reduce amphetamine, cocaine, and nicotine-induced locomotor stimulation, dopamine release, and CPP in rat tests (Ref. 148,149) (Figure 5). Moreover, numerous clinical and preclinical investigations have been carried out on various ghrelin receptor antagonists. For instance, YIL781 has been shown to enhance diabetic conditions by stimulating glucose-dependent insulin secretion and increasing body weight (Ref. 150), and PF-5190457 has been suggested as a potential pharmacological agent for the treatment of alcohol use disorders (Ref. 151). BIM28163 stimulates feeding and is clinically important in conditions such as cachexia or anorexia nervosa (Ref. 152) (Table 2).

**Table 2.** Ghrelin receptor antagonist showing therapeutic potential in preclinical and/or clinical studies

Agent	Target	potential therapeutic applications	Efficacy	Reference
LEAP–2	GHS-R1a	Obesity	Appetite suppression and metabolic remodeling	(143)
[D-Lys–3] GHRP–6	GHS-R1a	Alcoholic hepatic steatosis and alcohol abuse disorders	Decreasing hepatic steatosis and alcohol abuse	(145)
JMV2959	GHS-R1a	Drug addiction	Decreased locomotor stimulation, dopamine release, and CPP	(148, 149)
YIL781	GHS-R1a	Diabetes	Increases the production of insulin and weight gain	(150)
PF–5190457	GHS-R1a	Alcohol abuse	Reducing alcohol intake	(151)
BIM28163	GHS-R1a	Cachexia and anorexia.	Stimulated food intake and cFos activity	(152)

## Conclusions and perspectives

Fibrosis is a pathological process involving excessive ECM deposition and necrosis of organ parenchyma cells. It can lead to significant organ sclerosis as well as connective tissue proliferation and fibrosis. Tumor formation may result from fibrosis that continues to progress. The primary pathogenic process involves the transformation of fibroblasts into myofibroblasts, facilitated by the cytokine TGF-β. The use of TGF-β inhibitors for the treatment of fibrosis has shown promise in animal and clinical research (Refs. 153, 154). There is growing evidence that TGF-β functions as an effective therapeutic target in fibrotic diseases. Numerous approaches to treating fibrosis have been researched, including suppressing fibrosis to lower its propensity to become tumours and treating fibrotic disorders of the liver, kidney, lungs, and myocardium (Refs. 110, 155–157). Ghrelin is a potent fibrosis inhibitor. The antifibrotic activity of ghrelin is not well understood, and a better understanding of how ghrelin suppresses the fibrotic pathway could enhance its therapeutic efficacy. Ghrelin exerts anti-fibrotic effects by regulating inflammatory factors in different fibrotic diseases (Table 3). Previous studies indicate that ghrelin functions as an inhibitor of TGF-β. It also suppresses the expression of TGF-β, lowers the levels of Smad2/3, a signalling molecule downstream of TGF-β, and prevents Samd2 and Samd3 from becoming phosphorylated, all of which reduce tissue injury-induced fibrosis (Ref. 68). It was demonstrated that ghrelin inhibited TGF-β signalling through the typical Smad-dependent pathway. Therefore, this research presents the most recent data regarding the mechanism of ghrelin in treating fibrosis, suggesting that ghrelin can improve fibrotic diseases through TGF-β signalling. However, the complex biology of TGF-β poses significant challenges for clinical translation. This paper also covers the role of ghrelin receptor antagonists in obesity, anorexia, cachexia, metabolic diseases, and substance use disorders. This raises the question of whether ghrelin receptor antagonist use may invalidate ghrelin’s antifibrotic benefits and prompts research into how ghrelin receptor antagonists affect fibrotic disorders.

**Table 3.** Ghrelin regulates inflammatory cytokines in different diseases

Cytokine(s) regulated by ghrelin	Diseases	Mechanism/target	References
TGF-β, TNF-α,IL–1β, IL–6	Liver fibrosis	TGF-β1 activates both canonical and non-canonical pathways, including JNK, PI3K/AKT, and ERK	(8), (91), (97)
TNF-α	NASH	GHSR-dependent	(93)
TGF-β, PDGF-α, CTGF, COL–1, MMP2, MMP1	Cholestasis	AMPK pathway	(14)
TNF-α, IL–6	Sepsis	MAPK P38 pathway	(26)
TNF-α, α-SMA, SIRT–1, IL–1β, IL–6, ROS, PGC–1α, PAI–1	Kidney injury	GHSR-dependent	(109), (110), (113)
TGF-β, Smad3, α-SMA, COL–1	Kidney fibrosis	TGF-β/Smad3 pathway	(111)
GLP–1, GIP	Cystic fibrosis	NA	(112)
IL–6, Sirtuin1	Anovexia	NA	(114)
TNF-α,IGF–1, IL–1β	Myocardial infarction	NA	(33), (124)
LCβ–11,CD68, COLLa1, COLLa3	cardiac hypertrophy	CaMKK/AMPK signalling pathway	(125)
TGF-β GDF15,COL–1,COL–3	Myocardial fibrosis	TGF-β/Smad3 pathway NF-kB pathway	(34) (128)
NF-kB, IL–1β, IGF–1 TGF-β	Lung injury	NF-kB pathway miR–125a–5p/ ruppel-like factor 13 axis	(131) (133)
TGF-β, NF-kB, P65, P300	Skin injury	GHSR-dependent	(134)

Studies have indicated that chewing betel nuts significantly contributes to the development of oral submucous fibrosis (OSF). The primary mechanism of betel nut molecular regulation in OSF pathogenesis is through the activation of TGF-β and its downstream effectors, Smad2 and Smad3 (Ref. 158). This research investigates the antifibrotic effects of ghrelin, focusing primarily on the inhibition of TGF-β/Smad signalling. Ghrelin may thus be a potential treatment for OSF. However, its exact protective mechanisms and the associated histopathological changes still need deeper exploration. To mitigate the adverse effects of TGF-β in fibrotic diseases, more effective methods of inhibition should be explored. Future research should investigate ghrelin’s potential to specifically suppress TGF-β signal transduction, allowing TGF-β to retain its beneficial functions in human clinical treatment.

## Data Availability

All dates have been included in the manuscript.

## Abbreviations

AD, Atopic dermatitis; ADAMTS, A disintegrin and metalloproteinase with thrombospondin motifs; AG, Acylated ghrelin; ALT, Alanine aminotransferase; AngII, Angiotensin II; AST, Aspartate transaminase; ATP, Adenosine triphosphate; BDL, Bile duct ligation; BUN, Blood urea nitrogen; CKD, Chronic kidney disease; CPP, Conditioned position preference; CTGF, Connective tissue growth factor; CTHRC1, Collagen triple helix repeat containing 1; CVF, Collagen volume fraction; CVLM, audal ventrolateral medulla; DAG, Desacyl ghrelin; ECM, Extracellular matrix; EMT, Epithelial-mesenchymal transition; ER, Endoplasmic reticulum; ERK, Extracellular regulated protein kinases; GDF15, Growth differentiation factor 15; GH, growth hormone; GHS-R, Growth hormone secretagogue receptor; GHSR-1a, Growth hormone secretagogue receptor type 1a; GLS1, Glutaminase; GOAT, Ghrelin O-acyltransferase; GR, Glucocorticoid receptor; GSK-3β, Glycogen synthase kinase-3β; HCC, Hepatocellular carcinoma cell; HDAC3, Histone deacetylase 3; HSC, Hepatic stellate cell; ID1, DNA-binding protein inhibitor; IL-1β, Interleukin-1β; IPF, Idiopathic pulmonary fibrosis; ITG, Integrins; JNK, c-Jun N-terminal kinase; KP1, Klotho protein 1; LAP, Latency-associated peptide; LDH, Lactate dehydrogenase; LDTg, Laterodorsal tegmental area; LEAP-2, liver-expressed antimicrobial peptide 2; LLC, Large latent complex; LTBP, Latent TGF-β binding proteins; LV, left ventricular; MDA, Malondialdehyde; MFBs, Myofibroblasts; MI, Myocardial infarction; MMPs, Matrix metalloproteinases; MPO, Myeloperoxidase; N.Acc, Nucleus accumbens; NA, Nucleus ambiguous; NAFLD, Non-alcoholic liver disease; NAG, Neutral alpha-alucosidase; NCoR, Nuclear receptor corepressor; NE, Norepinephrine; NF-kB, Nuclear factor kB; NGAL, Neutrophil gelatinase-associated lipocalin; nGRE, Negative glucocorticoid response element; NOX, NADPH oxidase; NTS, Nucleus tractus solitarius; OSF, Oral submucous fibrosis; PAI-1, Plasminogen activator inhibitor-1; PC1/3, Prohormone convertase 1/3; PDGF-α, Platelet-derived growth factor-α; PGC-1a, Peroxisome proliferator-activated receptor-c coactivator 1a; PKCδ, protein kinase C; PVN, Paraventricular nucleus; QSG, Qishen granule; ROS, Reactive oxygen species; RVLM, Rostral ventrolateral medulla; SIRT1, Recombinant Sirtuin 1; SLC, Small latent complex; SSc, Systemic sclerosis; TCA, Tricarboxylic acid; TGF-1, Transforming growth factor-1; TGF-β, Transforming growth factor-beta; TIMP, Tissue inhibitor of metalloproteinase; TNF-α, Tumor necrosis factor-α; TSLP, Thymic stromal lymphopoietin; TSP-1, Thrombospondin-1; UCP2, Mitochondrial uncoupling protein; UUO, Unilateral ureteral obstruction; VTA, Ventral tegmental area.
